# Pleiotropic Function of Antenna-Specific Odorant-Binding Protein Links Xenobiotic Adaptation and Olfaction in *Leptinotarsa decemlineata*

**DOI:** 10.3390/insects16121259

**Published:** 2025-12-11

**Authors:** James A. Abendroth, Timothy W. Moural, Casey Cruse, Jonathan A. Hernandez, Michael S. Wolfin, Thomas Charles Baker, Andrei Alyokhin, Fang Zhu

**Affiliations:** 1 Department of Entomology, Pennsylvania State University, University Park, PA 16802, USA; jaa6479@psu.edu (J.A.A.); mvw5315@psu.edu (M.S.W.);; 2 School of Biology and Ecology, University of Maine, Orono, ME 04469, USA; alyokhin@maine.edu; 3 Huck Institutes of the Life Sciences, Pennsylvania State University, University Park, PA 16802, USA

**Keywords:** odorant-binding protein, olfaction, insecticide resistance, host location, sequestration

## Abstract

The Colorado potato beetle (CPB) is the primary defoliator of potatoes and is notorious for its ability to develop resistance to various insecticides. This remarkable adaptability may partly reflect selective pressures imposed due to the beetle’s coevolution with toxic Solanaceous host plants. As the initial interface between the environment and the insect olfactory system, odorant-binding proteins (OBPs) may sequester excess harmful molecules, such as insecticides and plant allelochemicals, in the perireceptor space, mitigating deleterious effects on vulnerable olfactory sensory neuronal dendrites. In this study, we identified an antenna-specific OBP (*LdecOBP33*) that is significantly upregulated in a pesticide resistant strain compared to a susceptible one. Competitive displacement fluorescence binding assays demonstrated that the LdecOBP33 protein exhibited broad affinity toward a range of plant volatiles and insecticides. Silencing *LdecOBP33* decreased the beetle’s resistance to imidacloprid and impaired its ability to locate host plants. Together, these findings provide insight into a key molecular factor involved in the CPB’s response to environmental challenges, suggesting a potential link between insects’ adaptation to xenobiotics and their olfactory processing.

## Simple Summary

*Leptinotarsa decemlineata*, known as the Colorado potato beetle (CPB), is renowned for its ability to adapt to adverse conditions. It has evolved resistance to every major class of insecticides, yet the mechanisms driving this adaptability remain unclear. In this work, we discovered that an odorant-binding protein (OBP) in the CPB mediates both imidacloprid resistance and host plant location, revealing a potential link between xenobiotic adaptation and olfaction.

## 1. Introduction

An essential determinant of insect success is their ability to recognize volatile chemical clues from potential hosts, conspecifics, and predators [1,2]. In terrestrial insects, volatile detection primarily occurs in the antennae, which are finely tuned and highly sensitive sensory structures that are specialized to fulfill this essential role [3,4]. Odorant-binding proteins (OBPs) are important components of this system; they act as the initial interface between the environment and the insect olfactory system [5]. Typically, OBPs selectively bind and solubilize hydrophobic volatile molecules, protecting them from degradation before reaching the respective olfactory receptor [6,7]. Recent evidence suggests that OBPs not only facilitate olfactory processing but also sequester excess harmful molecules in the perireceptor space, preventing them from reaching vulnerable olfactory sensory neuronal dendrites [3,8,9]. These molecules include plant volatiles, pheromones, and pesticides, all of which can be harmful to insect olfactory receptors when present in excess [5,10,11,12,13].

The Colorado potato beetle (CPB), *Leptinotarsa decemlineata*, is a notorious agricultural pest known for its ability to rapidly develop resistance to pesticides [14,15,16]. In less than a century since the first synthetic pesticide was introduced, CPB has evolved resistance to 57 different active ingredients, ranking among the 20 most pesticide-resistant arthropod pests [17,18,19]. It has been hypothesized that this ability is linked to the CPB’s history of coevolution with Solanaceous plants [20,21]. The CPB is specialized toward Solanaceous plants, which are commonly known as the nightshade family. This family includes key agricultural crops, such as potato, tomato, and eggplant, which contain high levels of plant allelochemicals across various parts of the plant [16,20,22,23,24]. Potato plants damaged by CPBs emit 7 to 10 times more herbivore-induced plant volatiles, such as linalool, 1-hexanol, (E)-2-hexenal, and 2-phenylethanol, than undamaged plants [25,26,27]. These volatiles encompass monoterpenoids, C_6_-aldehydes, or alcohols, which can exhibit antimicrobial activity at high concentrations, posing potential risks to adult CPBs’ antennae [25,26,28]. Despite this challenge, the CPB readily feeds on potato plant foliage at all developmental stages, indicating the presence of adaptive mechanisms that facilitate host plant utilization. While the previous literature has described how insects adapt to toxic allelochemicals in their food [29,30,31,32], little is known about how they cope with host plant volatiles.

In this study, seeking to bridge the above-mentioned knowledge gaps, we functionally characterized an antenna-specific OBP (*LdecOBP33*) that is highly expressed in adults, with particularly high expression in male antennae. The objective of this study was to elucidate the functional roles of *LdecOBP33* in the CPB’s adaptation to xenobiotics and host plant cues. Specifically, we evaluated the binding affinity of recombinant LdecOBP33 toward diverse potato volatiles and multiple classes of insecticides. Moreover, we used RNAi feeding to determine how *LdecOBP33* silencing affects imidacloprid resistance and host plant location in adult beetles. Although 59 OBPs have recently been identified in the CPB genome, none have been functionally characterized [33]. By integrating molecular, biochemical, and behavioral approaches, this study serves to advance our understanding of the potential contribution of olfactory proteins to the exceptional adaptability of the CPB.

## 2. Materials and Methods

### 2.1. Insects

Two different populations of CPBs were used for this study. The susceptible CPB population was purchased from French Agricultural Research (Lamberton, MN, USA). This population was initially collected in 2003 from Long Island, NY; following this, it was reared in laboratory conditions with no pesticide exposure. Neonicotinoid-resistant CPBs were collected from the University of Maine Aroostook Research Farm (Presque Isle, ME, USA) [34]. Baseline mortality exposure to imidacloprid in adults indicated a ~30-fold resistance ratio between the resistant and susceptible strains. Both CPB populations were reared in a Penn State facility greenhouse at 25 ± 1 °C under a 16:8 L/D photoperiod. Beetles were fed a constant supply of Red Norland potato plants and housed in insect and butterfly habitat cages (Restcloud, Guangzhou, China). Eggs were collected from plants and stored in Petri dishes maintained at 25 ± 1 °C and 60 ± 5% humidity under a 16:8 L/D photoperiod, located within a rearing room. Newly emerged first-instar larvae fed upon fresh potato plant leaflets up to the third instar and were then transferred back to the greenhouse.

### 2.2. In Silico Structural and Phylogenetic Analyses

*LdecOBP33* was the top candidate identified in our previous transcriptomics study of CPB antennae and exhibited 100% sequence similarity to XM_023165485 [24,33]. The signal peptide of LdecOBP33 was determined using SignalP 6.0 (https://services.healthtech.dtu.dk/services/SignalP-6.0/, accessed on 2 November 2025). The isoelectric point (pI) and molecular weight (mW) were estimated using ExPASy proteomic tools (http://web.expasy.org/compute_pi, accessed on 2 November 2025). Secondary and tertiary structures were predicted for LdecOBP33 using AlphaFold 2 [35,36]. In total, 219 OBP sequences from 5 insect species (*Leptinotarsa decemlineata*, *Tribolium castaneum*, *Diabrotica virgifera virgifera, Monochamus alternatus*, and *Collaphellus bowringi*) were obtained from the NCBI database and previously published studies (Table S1). Multiple sequence alignment was performed using MUSCLE in MEGA 11 v11.0.13 [37], using the default parameters. The maximum likelihood tree was generated in MEGA 11 v11.0.13 using the LG + G + I model with 1000 bootstraps [5].

### 2.3. RNA Extraction, cDNA Synthesis, and qRT-PCR

Total RNA extraction was conducted using 3–50 CPBs from each life stage: one-day-old eggs (50); five-day-old eggs (50); larvae at the first (10), second (6), third (4), and fourth (3) instars; pupae (3); and one-week-old adult males (3) and females (3). Additionally, specific tissue was collected from one-week-old adult males and females, which included the antennae, head, legs, midgut, fat bodies, Malpighian tubules, and sex organs. Once collected, tissue was homogenized in TRIzol^®^ reagent (Thermo Fisher Scientific, Waltham, MA, USA) according to the manufacturer’s instructions. RNA samples were then treated with Invitrogen Turbo^TM^ DNAse (Thermo Fisher Scientific, Waltham, MA, USA) to eliminate genomic DNA contaminants. Purified RNA samples were then used to create transcript cDNA with M-MLV reverse transcriptase (Promega, Madison, WI, USA). A NanoDrop One (Thermo Scientific, Madison, WI, USA) was used to measure the concentration of cDNA. The qRT-PCR was carried out using a CFX96 Touch Deep Well Real-Time PCR Detection System (Bio-Rad Laboratories, Hercules, CA, USA). The total reaction volume was 10 µL, which included 1 µL cDNA, 5 µL Forget-Me-Not^TM^ EvaGreen qRT-PCR Master Mix (Avantor Inc., Radnor, PA, USA), 0.4 µL qRT-PCR primer (Table S2), and 3.6 µL ddH_2_O. The program used for all reactions consisted of initial incubation at 95 °C for 3 min, 40 cycles at 95 °C for 10 s, 55 °C for 30 s, 95 °C for 10 s, and lastly 65 °C for 5 s. Elongation factor 1α (*EF1α*) and ribosomal protein L4 (*RPL4*) were used as reference genes to normalize the threshold cycle (Ct) values; this was based on our previous studies [38,39]. Relative gene expression was determined with the 2^−ΔΔCt^ method [40]. Three biological replications and two to four technical replications were conducted independently.

### 2.4. RNA Interference (RNAi)

The specific dsRNA of *LdecOBP33* was prepared using an Invitrogen MEGAscript^TM^ T7 transcription kit (Thermo Fisher Scientific, Waltham, MA, USA) with the primers listed in Table S2. The dsRNA of a green fluorescent protein (GFP) gene (*dsGFP*) was generated using a pET His6 GFP TEV LIC cloning vector plasmid (addgene#29663) as a template. The reaction consisted of an initial incubation period at 37 °C for 6 h, followed by an extension step at 75 °C for 5 min, and finally an overnight annealing step at room temperature. Then, the dsRNA was purified using an Invitrogen Turbo DNA-*free*^TM^ Kit (Thermo Fisher Scientific, Waltham, MA, USA). The quality and length of dsRNA were assessed through both agarose gel electrophoresis and a NanoDrop One Microvolume UV–Vis spectrophotometer (Thermo Fisher Scientific, Waltham, MA, USA). The RNAi feeding procedure for dsRNA delivery to CPB adults was adapted from our previous studies [38,39]. In brief, one-week-old adult male or female CPBs were fed 3 µg dsRNA of *LdecOBP33* or *GFP* (control) per individual on a potato leaf disc after 24 h of starvation. The treatment lasted three days, after which beetles were fed untreated potato leaf discs for an additional two days. On the sixth day, CPBs were collected for either knockdown efficiency evaluation via qRT-PCR or toxicology assays, or they were starved for an additional 24 h for use in behavioral bioassays.

### 2.5. Heterologous Expression and Purification of LdecOBP33

A pET28a (+) plasmid with codon-optimized full-length *LdecOBP33* (XM_023165485) inserted between the NdeI and HindIII cut sites was ordered from GenScript^TM^. Then, the signal peptide and thrombin cut site were removed using a New England Biolabs Q5^®^ site-directed mutagenesis kit with the deletion primers listed in Table S2, resulting in the final expression construct. The final plasmid construct was sequenced via Plasmidsaurus^TM^ (Arcadia, CA, USA) to confirm its accuracy. Then, the plasmid was transformed into the Shuffle^®^ T7 lysY-competent *E. coli* expression strain (NEB, Ipswich, MA, USA) for expression. Shuffle^®^ cells were selected rather than standard BL21 strains in order to express recombinant LdecOBP33 in the soluble fraction, avoiding the labor-intensive refolding of inclusion bodies. This strain is engineered to have an enhanced ability to express correctly folded proteins with multiple disulfide bonds in the cytoplasm. Shuffle^®^ cells containing pET28a-LdecOBP33 were incubated at 30 °C overnight at 250 rpm, suspended in 50 mL of 2x yeast extract tryptone medium and 100 µg/mL of kanamycin. After 16–18 h, the culture was removed from the shaking incubator and used to inoculate 1.2 L of terrific broth medium supplemented with 100 µg/mL of kanamycin. Next, the expression culture was incubated at 30 °C at 250 rpm until it reached an optical density (OD_600 nm_) of between 0.7 and 0.9; after this, the culture was removed and chilled on ice for fifteen minutes, followed by induction with 1 mM of IPTG. The culture was then placed in a shaking incubator for 24 h at 16 °C and 250 rpm. Finally, the cell pellet was harvested and stored at −20 °C until protein purification.

For LdOPB33 purification, the cell pellet was resuspended in a buffer solution containing 50 mM NaPi, 500 mM NaCl, 10 mM imidazole, 3.3 mM NaN_3_, and 100 mM PMSF, adjusted to pH 7.4. A Pierce Protease EDTA-free inhibitor tablet (Thermo Scientific) was also added. The cells were then lysed via sonication (Branson Digital Sonifier SFX 150, Brookfield, CT, USA) on ice at 70% power for 30 s. The lysate was then centrifuged at 18,000 rcf for 15 min at 4 °C. The supernatant was transferred to a Kontes^®^ Flex-Column^®^ gravity column with a 5 mL Ni-NTA resin bed and washed with ten column volumes (CVs) of lysis buffer, followed by five CVs of 50 mM NaPi, 500 mM NaCl, 40 mM imidazole, and 3.3 mM NaN3, with a pH of 7.4. Then, the LdecOBP33 protein was eluted with eight CVs of 50 mM NaPi, 300 mM NaCl, 250 mM imidazole, and 3.3 mM NaN3, at a pH of 7.4. Then, it was buffer exchanged 100-fold for 20 mM MES and 1 mM EDTA, with a pH of 6.5, and injected onto a 5 mL HiScreen^TM^Capto^TM^ SP ImpRes chromatography column (Cytiva, Marlborough, MA, USA) connected to a Bio-Rad NGC (Bio-Rad Laboratories, Hercules, CA, USA). Then, a 20 CV gradient with buffer A (20 mM MES, 1 mM EDTA, pH 6.5) to buffer B (20 mM MES, 1 M NaCl, 1 mM EDTA, pH 6.5) was used to elute the LdecOBP33 protein. Next, dialysis was performed twice for 12 h each against 20 mM Tris and 5% methanol at pH 7.4 to remove potential endogenous ligands that could interfere with the binding ability [41,42]. An analysis of the chromatography fractions and final purified protein was performed with SDS-PAGE to assess the quality and quantity of the LdecOBP33 protein. Finally, the protein was flash-frozen with liquid nitrogen and stored at −80 °C for later use.

### 2.6. Fluorescence Binding Assay

N-phenyl-1-naphthylamine (1-NPN) was used as a fluorescent reporter to assess the ligand-binding affinity of LdecOBP33 toward various plant volatiles and pesticides; this was chosen due to its reported success in prior studies [43,44]. Firstly, to assess the binding affinity of LdecOBP33 to 1-NPN, a saturation binding assay was conducted with a constant protein concentration of 2 µM, while the concentration of 1-NPN varied between 0 µM and 25 µM; both were applied in 20 mM Tris at pH 7.4. To assess the binding affinities of plant volatiles and pesticides toward LdecOBP33, competitive fluorescence displacement assays were conducted with the LdecOBP33 protein at a constant concentration of 2 µM, 1-NPN at 2 µM, and competitor ligands at concentrations varying between 0 µM and 450 µM, all using 20 mM Tris at pH 7.4. Each assay was performed at 25 ± 1 °C in a 96-well flat-bottom black plate with a final volume of 200 µL and 3 replicates. The relative fluorescence intensity was measured with a 337 nm/10 nm excitation filter and a 413 nm/10 nm emission filter, using a multi-mode Tecan Spark^®^ plate reader. GraphPad Prism 8.0 was used to fit the saturation binding curve and to calculate the dissociation constant between LdecOBP33 and 1-NPN, and fluorescence inhibition curves were applied to calculate IC_50_ values for competitor ligands. The equation $Ki=\frac{\left[{IC}_{50}\right]}{\left(1+\left[NPN\right]/K_{d}NPN\right)}$ was used to calculate the *K_i_* values of the competitor ligands [45].

### 2.7. LdecOBP33 Protein Model and Ligand Docking

The LdecOBP33 protein model was predicted with AlphaFold 2 using ColabFold v1.5.5 with MMseqs2 [35,36,46]. Default settings were used, with the following modifications: relaxation was set to 1, the template was set to pdb100, the number of recycles was set to 12, the mxa_msa value was set to 32:64, and num_seeds was set to 2. The full length minus its signal peptide was used for the AlphaFold 2 model. The LdecOBP33 model was superposed with protein–ligand-complexed crystal structure homologs that had been identified with HHpred (PDB IDs: 3R72, 8BXW, 100H); this was used to identify the presumptive ligand-binding pocket [46,47,48]. The predicted structural homologs had HHpred probability scores of 99.89%, 99.9%, and 99.91%, respectively. Based on structural alignment with the AgamOBP5 (PDB ID 8BXW) co-crystal structure with carvacrol, the C-terminal loop of the LdecOBP33 AlphaFold 2 model was remodeled from Ile 131 to Pro 142 with MODELLER and then minimized with Amber ff14SB implemented in Chimera v1.15 [48,49,50,51]. Following this, molecular docking was performed using the DockingPie 1.2 plugin installed in PyMOL 3.0.4. Search exhaustion was set to 20, and the number of possible poses was increased to 3. The grid box was set to 20 Å, cubed and centered about the presumptive ligand-binding pocket. Docking was carried out using the Vinardo scoring function implemented via Smina [52,53]. The analysis of LdecOBP33 and docking was performed with Chimera, ChimeraX, and PyMOL; the resulting figures were constructed with ChimerX v1.8 [51,53,54,55,56].

### 2.8. Whole-Antenna Contact Bioassay with Imidacloprid

After feeding with RNAi, the control or *LdecOBP33* knockdown male CPBs were placed individually into fresh Petri dishes and anesthetized on ice for 10 min. Afterwards, 0.5 µL of an imidacloprid solution (Sigma Aldrich, St. Louis, MO, USA)—at various concentrations—in acetone was topically applied to each antenna of each CPB using a Hamilton 25 µL model #702 syringe (Hamilton, Reno, NV, USA). Care was taken to ensure that the pesticide solution was applied exclusively to the antennae. The selection of a 0.45 µg/µL dose of imidacloprid in acetone was based on preliminary tests, which indicated an LD_50_ of 0.45 µg/µL for a 0.5 µL application. After treatment, all CPBs were placed in a fresh Petri dish with fresh potato leaves; the dish was kept in a rearing room maintained at 25 ± 1 °C, 60 ± 5% humidity, and a 16:8 L/D photoperiod. CPB mortality was assessed at 0, 3, 6, and 12 h, and every 12 h thereafter, for a total of five days post-treatment. The determination of recovery for individual CPBs after pesticide treatment was adapted from prior studies [57,58] using three criteria. First, the hind leg was pinched; beetles that failed to respond were considered dead. Second, beetles were placed prone and given two minutes to flip upright; those unable to do so were classified as dead. Finally, beetles were placed upside down on the handle of a paintbrush to assess their mobility; individuals that could not walk their full body length or fell off were considered dead. Six biological replicates were conducted, each consisting of 8–10 beetles. No mortality was observed in beetles treated with acetone alone.

### 2.9. Behavioral Assay

The ability of CPB adults to locate a host plant was observed in behavioral assays. CPB adults were starved for 24 h following dsRNA feeding. Beetles were allotted one hour to acclimate to the room while within fresh Petri dishes prior to testing. An aluminum foil sheet measuring 30.50 cm × 40.65 cm served as an insert, forming a uniform arena in a wind tunnel, with a constant air flow rate of 1.5 m/s, a temperature of 22 ± 5 °C, and approximately 40–50% humidity. A potato leaf from a CPB larva-infested plant served as the odor stimulus and was placed at the upwind edge of the arena, along with a water-saturated cotton ball to maintain constant humidity [28,59]. Leaves were replaced with fresh infested leaves every 30 min. For the behavior assay, an individual CPB was gently placed at the opposite end of the arena to the potato leaf, facing upwind. Beetles that walked out of the arena were gently returned to the starting point. The time taken for each beetle to contact the leaf, covering a maximum of 5 min, was recorded. After the conclusion of each assay, CPBs were returned to a fresh Petri dish, and the tin foil arena was sanitized with a 70% ethanol solution. In total, two tin foil arenas were used for these bioassays. Control (ds*GFP*) and *LdecOBP33* knockdown beetles were tested alternately to minimize temporal variation.

### 2.10. Statistical Analysis

All data were first assessed for normality using the Shapiro–Wilk test. Data were then inspected regarding distribution and symmetry. For comparisons between two groups, Student’s *t*-test was used when data were approximately normally distributed and comprised no extreme outliers; otherwise, the non-parametric Mann–Whitney U test was applied. For comparisons involving multiple groups, one-way ANOVA followed by Tukey’s HSD post hoc test was used for roughly normally distributed data, whereas the non-parametric Kruskal–Wallis test followed by Dunn’s multiple comparisons was applied for non-normally distributed data. Differences between the control and *LdecOBP33* knockdown beetles regarding their ability to locate the host and resist pesticide treatment were analyzed using chi-squared tests. To compare the mortality of control and *LdecOBP33* knockdown beetles in response to imidacloprid over time, a survival analysis was performed using the Log-Rank and Wilcoxon tests. All statistical analyses were conducted using JMP v 17.0 (SAS Institute, Cary, NC, USA).

## 3. Results

### 3.1. Expression Patterns of LdecOBP33 and Bioinformatic Analyses

**Figure 1:**
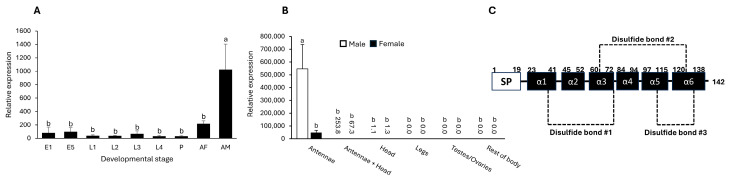
*LdecOBP33* expression profile and predicted protein secondary structure. (**A**) Developmental expression pattern. E1, one-day-old egg; E5, five-day-old egg; L1-L4, first- to fourth-instar larvae; P, pupae; AF, adult female; AM, adult male. (**B**) Spatial expression pattern. Rest of body refers to remaining body tissues, excluding antennae, head, legs, and reproductive organs. (**C**) Schematic diagram of secondary structure of LdecOBP33. SP, signal peptide; α1-α6, alpha helices 1–6; dashed lines indicate disulfide bond bridges. Data shown are mean ± SE (*n* = 6 or 9). Different letters indicate significant differences relative to *LdecOBP33* gene expression among developmental stages and tissues at *p* < 0.05 (Kruskal–Wallis test followed by Dunn’s multiple comparisons).

Developmental expression analyses showed that *LdecOBP33* was expressed across all development stages, with predominant expression in adults, and the highest expression was observed in male CPBs (Figure 1A). The spatial expression pattern of *LdecOBP33* indicated antenna-specific expression, with particularly high levels in male antennae (Figure 1B).

LdecOBP33 shared 100% sequence identity with an uncharacterized *Leptinotarsa decemlineata* protein (NCBI accession number: XM_023165485). Additionally, the predicted secondary and tertiary structures of LdecOBP33 featured the hallmark characteristics of a classic OBP, with six highly conserved cysteine residues and three disulfide bonds (Figure 1C). The molecular weight of LdecOBP33 was estimated to be at 15.259 kDa, with an isoelectric point of 8.18. A signal peptide made of amino acid residues 1–19 was detected as well, which was removed prior to protein expression.

To investigate the evolutionary relationships between LdecOBP33 and other Coleopteran OBPs, we performed a phylogenetic analysis. It was found that LdecOBP33 resided in one of the two classic OBP clades, alongside two other OBPs with broad binding affinities: MaltOBP10 [60] and MaltOBP13 [61] (Figure S1). Specifically, LdecOBP33 was clustered together with other classic insect OBPs that resided in an additional subfamily, termed antenna-binding protein II (ABPII) [62,63,64].

### 3.2. Binding of LdecOBP33 with Various Plant Volatiles and Pesticides

**Figure 2:**
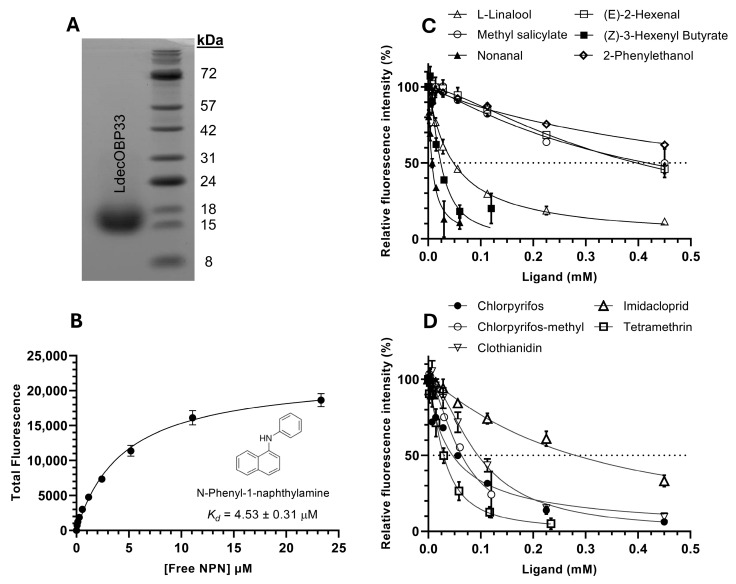
Competitive binding curves of plant compounds and pesticides regarding purified recombinant LdecOBP33 protein. (**A**) Binding affinity of purified recombinant LdecOBP33 protein toward fluorescent reporter 1-NPN, with dissociation constant (*Kd*) indicated. (**B**,**C**) Competitive binding assay inhibition curves of potato plant compounds and (**D**) pesticides regarding LdecOBP33. Data shown are mean ± SD (*n* = 3). The 50% fluorescence intensity threshold is shown as a dashed line.

To assess the binding affinity of LdecOBP33 toward potential ligands, we performed competitive fluorescence displacement assays with purified LdecOBP33 (Figure 2A) and various potato plant volatiles and pesticides. Firstly, using the highly purified LdecOBP33 protein, we determined the affinity of the fluorescent reporter 1-NPN through a non-linear regression one-site saturation binding curve that accounted for ligand depletion [65], which resulted in a dissociation constant of 4.53 ± 0.31 µM (Figure 2B). Secondly, the inhibition of the 1-NPN–LdecOBP33 complex in the presence of competitor ligands was determined using a non-linear inhibition curve fit model to obtain the respective IC_50_ (half-maximal inhibitory concentration (Figure 2A)) for each competitor ligand. For plant volatiles, we observed over 50% displacement of the 1-NPN probe with nonanal (IC_50_ = 7.29 ± 0.53 µM), (Z)-3-hexenyl-butyrate (IC_50_ = 23.99 ± 2.05 µM), L-linalool (IC_50_ = 48.26 ± 1.77 µM), methyl salicylate (IC_50_ = 396.30 ± 19.97 µM), and (E)-2-hexenal (IC_50_ = 401.40 ± 16.29 µM) (Table 1, Figure 2C). For the pesticides tested, over 50% displacement of the 1-NPN probe was observed with tetramethrin (IC_50_ = 29.07 ± 1.78 µM), chlorpyrifos (IC_50_ = 45.81 ± 3.53 µM), chlorpyrifos-methyl (IC_50_ = 63.67 ± 3.32 µM), clothianidin (IC_50_ = 94.51 ± 4.24 µM), and imidacloprid (IC_50_ = 262.80 ± 15.17 µM) (Table 1, Figure 2D). This indicates that this OBP has a relatively broad binding spectrum. No noticeable displacement of the 1-NPN probe in the presence of (E)-3-hexen-1-ol, D-glucose, or carbaryl was observed (Table 1).

Using the respective IC_50_ value obtained for each competitor ligand, the inhibitory constant (Ki) was calculated with the Cheng and Prusoff equation [66]. Regarding plant volatiles, the calculated Ki values were 5.47 µM for nonanal, 18.01 µM for (Z)-3-hexenyl-butyrate, 36.23 µM for L-linalool, 297.54 µM for methyl salicylate, 301.37 µM for (E)-2-hexenal, and 629.54 µM for 2-phenylethanol. Regarding pesticides, the calculated Ki values were 21.83 µM for tetramethrin, 34.39 µM for chlorpyrifos, 47.80 µM for chlorpyrifos-methyl, 70.96 µM for clothianidin, and 197.31 µM for imidacloprid (Table 1).

### 3.3. Structure Modeling and Molecular Docking with Nonanal and Imidacloprid

**Figure 3:**
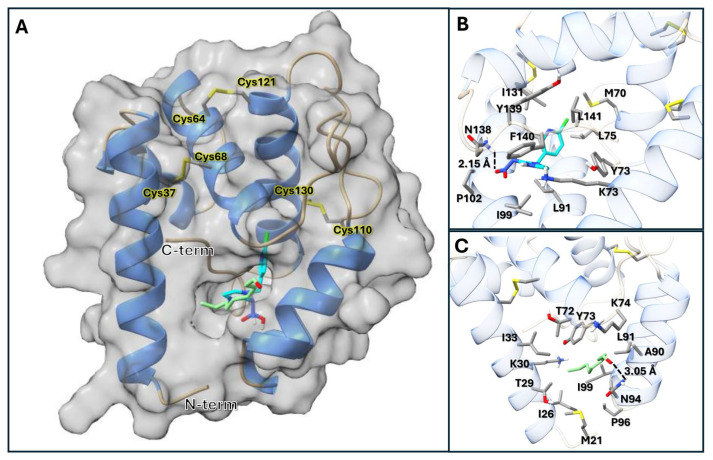
AlphaFold 2 model of LdecOBP33. (**A**) Global fold of LdecOBP33 displayed as ribbon diagram (helices in blue and coils in tan) overlaid with transparent surface showing ligand-binding pocket. Conserved residues are shown and labeled with respective cysteine residues. (**B**) Highest-scoring pose of imidacloprid (indicated by heteroatom, with carbons in cyan); all sidechains within 4 Å are displayed and labeled. (**C**) Highest-scoring pose of nonanal (indicated by heteroatom, with carbons in green); all sidechains within 4 Å are displayed and labeled.

The AlphaFold 2 model of LdecOBP33 revealed a compact bundle of six α-helices surrounding a hydrophobic ligand-binding pocket (Figure 3A). The fold is stabilized by three conserved disulfide bonds, formed by Cys37-Cys68, Cys64-121, and Cys110-Cys130. Structural alignment with homologs identified the ligand-binding pocket. As depicted in Figure 3A, the ligand-binding pocket opens near the N-terminus and extends as a channel running toward the C-terminus. While many residues lining the pocket are hydrophobic, some polar and charged residues are also present and are expected to play key roles in hydrogen bonding with ligands. The structural homologs predicted with HHpred, namely 3R72, 8BXW, and 100H, had homolog probability scores of 99.89%, 99.9%, and 99.91%, respectively. Molecular docking with nonanal and imidacloprid revealed two key residues potentially involved in hydrogen bond formation. In the highest-scoring pose (−5.9 kcal/mol) of imidacloprid, a hydrogen bond (2.15 Å) between Asn138 HD2 and the O2 atom of imidacloprid was observed (Figure 3B). Additionally, in the top-ranking pose of nonanal (−5.4 kcal/mol), the carbonyl oxygen formed a hydrogen bond (3.05 Å) with the HD21 hydrogen of Asn94 (Figure 3C). Docking was also performed for two additional ligands, chlorpyrifos (−5.2 kcal/mol) and tetramethrin (−9.3 kcal/mol).

### 3.4. Contribution of LdecOBP33 to Imidacloprid Resistance in CPB

**Figure 4:**
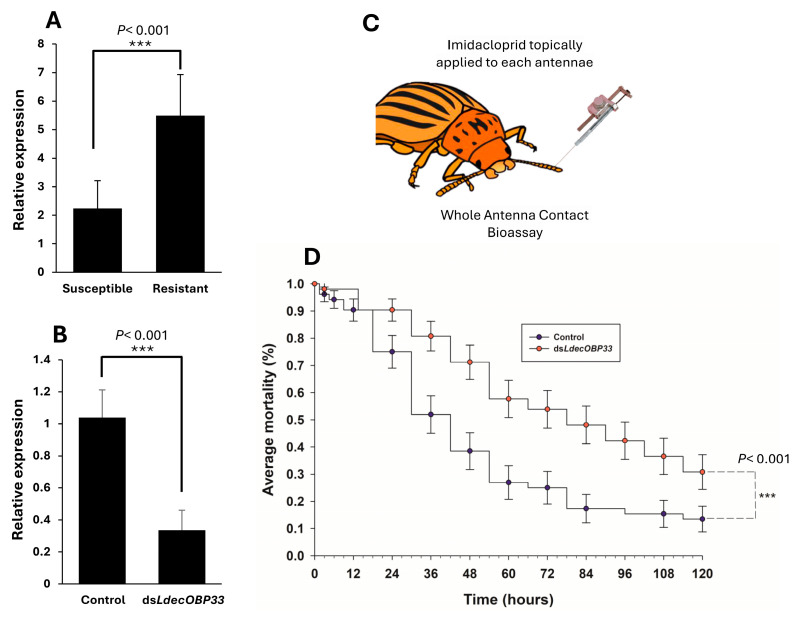
Influence of *LdecOBP33* on pesticide resistance in adult male CPB. (**A**) Differential expression of *LdecOBP33* in susceptible and resistant CPB strains. Data shown are mean ± SE (*n* = 4). (**B**) Relative expression of *LdecOBP33* in CPBs fed ds*GFP* (control) and ds*LdecOBP33*. Statistical significance is denoted by ‘***’ for *p*-values < 0.001, based on Student’s *t*-test. Data shown are mean ± SE (*n* = 6). (**C**) Illustration of whole-antenna contact bioassay. CPBs were anesthetized on ice for 10 min, after which 0.5 µL of 0.45 µg/µL imidacloprid solution was topically applied to each antenna. (**D**) Survival was observed across 120 h. Data shown are mean ± SE (*n* = 6). Statistical significance is denoted by ‘***’ for *p*-values < 0.001, based on Log-Rank and Wilcoxon tests.

To determine whether *LdecOBP33* was associated with resistance, we compared its expression between susceptible and imidacloprid-resistant CPB strains. We found that *LdecOBP33* expression was 2.5-fold higher in the resistant strain compared to the susceptible one (Figure 4A). To determine whether *LdecOBP33* contributed to imidacloprid resistance in CPBs, we first silenced *LdecOBP33* expression in imidacloprid-resistant CPB males through RNAi feeding. After 5 days, *LdecOBP33* had yielded a 77% reduction in transcriptional expression in *dsLecOBP33*-feeding beetles compared to ds*GFP*-feeding beetles, confirming the successful silencing of *LdecOBP33* (Figure 4B). Next, we conducted an antenna contact bioassay by applying imidacloprid to both antennae in CPB males (Figure 4C) and monitored their mortality over five days. During the first 36 h, there was no significant difference in mortality between the *LdecOBP33* silencing group and the ds*GFP* control group (Figure 4D). However, after 36 h, ds*LdecOBP33*-KD beetles showed significantly higher mortality after exposure to imidacloprid (81%) compared to control beetles (55%) (Figure 4D), indicating a general decrease in survival. By 120 h, the average mortality in ds*LdecOBP33*-treated CPBs was 32%, while that in control beetles was 12%, indicating an increase of 19% (Figure 4D).

### 3.5. Roles of LdecOBP33 in Host Location

**Figure 5:**
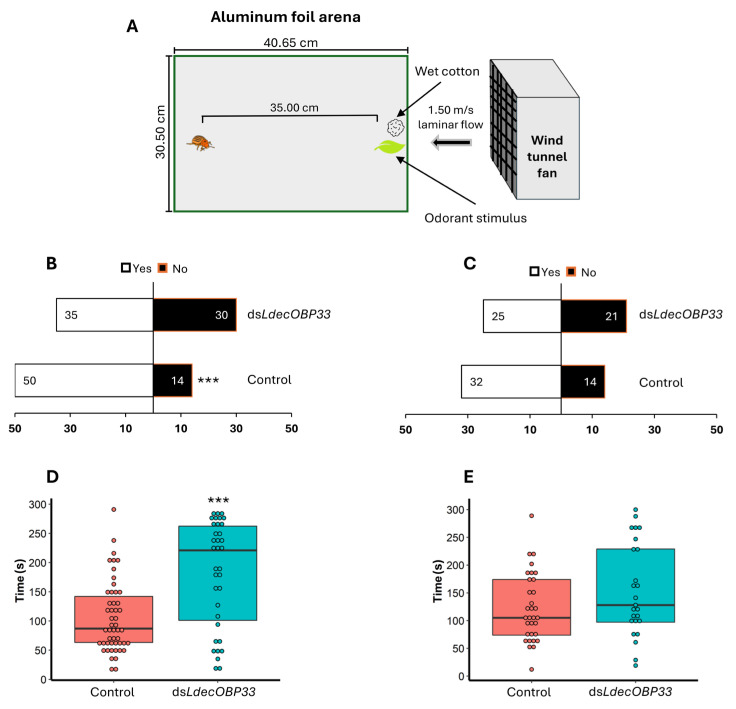
Effects of *LdecOBP33* on host plant location behavior in adult CPB. (**A**) Schematic of behavioral assay setup. CPBs were given five minutes to locate potato leaf tissue. Differences in ability of adult male (**B**) and female (**C**) CPBs to locate potato leaves between control (ds*GFP*) and *LdecOBP33*-silenced individuals. Differences in time taken for adult male (**D**) and female (**E**) CPBs to successfully locate potato leaves between control (ds*GFP*) and *LdecOBP33*-silenced individuals. Statistical significance is denoted by ‘***’ for *p*-values < 0.001, based on chi-squared test (**B**,**C**) and Mann–Whitney U test (**D**,**E**). In (**D**), controls consisted of 50 males, while 35 were used for *LdecOBP33* silencing. In (**E**), controls consisted of 32 females, while 25 were used for *LdecOBP33* silencing.

We compared the responses of *LdecOBP33-*KD and control beetles to potato leaves infested with CPB larvae in a wind tunnel (Figure 5A). We recorded two key aspects: (1) whether beetles could locate the host odor source (an excised leaf) within the 5 min test period and (2) the time that was taken to achieve contact with the leaf. After *LdecOBP33* was silenced, the host location ability in adult male CPBs declined. Specifically, significantly fewer male beetles in the *LdecOBP33*-silenced group successfully located the potato leaf within the 5 min test period as compared to control beetles (Figure 5B). Among the beetles that reached the potato leaf within 5 min, *LdecOBP33*-silenced male beetles required significantly longer than control beetles (Figure 5D). On average, *LdecOBP33*-silenced male beetles achieved contact with the leaf in 205 s (95% CI: 176 to 234 s), whereas control beetles required 106 s (95% CI: 81 to 132 s) (Figure 5D).

## 4. Discussion

We functionally characterized an antenna-specific OBP in the CPB, revealing that it acts as both a transporter of odorant signals and a scavenger of hydrophobic molecules, including both plant volatiles and insecticides. Thus, a link between olfaction and xenobiotic adaptation was established. *LdecOBP33* exhibits the highest expression in the antennae of male CPBs and belongs to the subfamily of antenna-binding protein II (ABPII), a group of classic OBPs (Figure S1). In general, recombinant LdecOBP33 displayed a broad binding spectrum similar to those of many other classic insect OBPs [67,68,69]. LdecOBP33 showed a preference for binding to the most hydrophobic molecules tested in this study, following a general trend of increasing affinity to a molecule as the lipophilicity (LogP) increased (Table 1). Traditionally, OBPs have been understood to play a key role in transporting exogenous hydrophobic molecules through the aqueous sensillar lymph [1,3,70]. However, recent research strongly suggests that OBPs also function as scavengers or buffering agents, interacting with excess molecules in the perireceptor space to remove insoluble ligands or modulate their concentrations before they reach the olfactory receptors [3,12,13,71]. Although plant volatiles serve as key ecological signals in insect host location, they can pose physiological risks at high concentrations, potentially leading to sensory overstimulation or neurotoxicity [3,5,72]. The outcomes of our binding assays suggest that LdecOBP33 exhibits a relatively broad affinity for hydrophobic molecules, such as potato volatiles and pesticides, potentially facilitating their rapid processing in the perireceptor space and protecting the insect nervous system from harmful effects.

Serving as the initial interface between the environment and the insect olfactory system, OBPs solubilize and transport hydrophobic odorant molecules through the sensillar lymph to olfactory receptors [1]. Pesticides may penetrate the antennal lymph and be sequestered by OBPs, preventing their interaction with target proteins and mitigating potential neurotoxic effects [73]. Thus, it is hypothesized that OBPs contribute to insecticide resistance [5,73]. In this study, the differential gene expression analysis of *LdecOBP33* revealed 2.5-fold higher expression in a neonicotinoid-resistant strain in comparison to the susceptible strain. The RNAi-mediated gene silencing of *LdecOBP33* led to increased imidacloprid susceptibility in resistant beetles, suggesting that *LdecOBP33* contributes to resistance. The delayed rise in mortality at 36 h after *LdecOBP33* knockdown is consistent with a sequestration-based mechanism. Recent research has shown that chemosensory proteins can contribute to insecticide resistance, suggesting a broader role for insect OBPs and related chemical-binding proteins in detoxification [3,73,74]. Moreover, the RNAi-mediated silencing of specific OBPs has been associated with increased insect susceptibility to insecticides [75,76]. For example, in *Anopheles gambiae* mosquitoes, a sensory appendage protein, SAP2—which is highly expressed in the legs of pyrethroid-resistant populations binds pyrethroids and contributes to pyrethroid resistance [74]. Another example is the brown planthopper *Nilaparvata lugens*, in which the OBP *NIOBP3* is constitutively overexpressed in strains resistant to nitenpyram and sulfoxaflor [76]. Silencing of *NlOBP3* significantly increased the susceptibility of *N. lugens* to both insecticides, suggesting that *NlOBP3* is associated with resistance to nitenpyram and sulfoxaflor [76].

It is understood that the behavioral adaptation of an insect to its host can be influenced by the evolution of its chemosensory system [77,78,79,80]. In comparison to other chemosensory gene families, OBPs are highly divergent in both form and function, exhibiting low sequence similarity even within members of the same species [5,81]. Due to their important roles as the primary mediators between the external environment and the insect olfactory system, OBPs may play an important role in the adaptation of insects to their host plants. Prior studies have observed that CPBs can discriminate their preferred host plant, potato, from other solanaceous species by detecting a specific ratio of volatile compounds that are unique to potato plants [82,83,84]. Disruption of the CPB’s ability to successfully perceive odor blend ratios in potato can impair its ability to locate its host plant. In this work, we found that, after the knockdown of *LdecOBP33*, adult male CPBs required significantly longer periods of time to locate the host plant in comparison to the control group. These findings are consistent with previous studies showing that the RNAi-mediated gene silencing of insect OBPs influences host location [85,86,87].

Additionally, recombinant LdecOBP33 exhibited binding affinity toward a variety of potato plant volatiles. Among these volatiles, the strongest affinity for LdecOBP33 was observed for nonanal, (Z)-3-hexenyl-butyrate, and L-linalool. In addition, binding was observed for two other volatiles, methyl salicylate and (E)-2-hexenal, as they were able to displace 1-NPN by over 50% (Table 1, Figure 2C). A similar phenomenon was also observed in recent studies [86,88]. (Z)-3-Hexenyl-butyrate and methyl salicylate are plant-emitted volatiles that are typically released under stress and have been shown to attract CPBs and elicit antennal responses [28,89]. Moreover, methyl salicylate has been used in several synthetic cocktails that have elicited attractive responses from CPBs [90,91]. However, there may not be a direct correlation between the binding affinity of a singular OBP for an odorant and the odorant’s ability to induce olfactory activity, as the antennal response to a volatile can depend on the cooperation of one or more OBPs [1,92,93]. Nonetheless, the broad binding affinity of LdecOBP33 toward potato plant volatiles, alongside the findings of our behavioral assays, suggests that LdecOBP33 plays a fundamental role in olfactory processing in the adult male CPB.

*LdecOBP33* was originally termed OBP10 in early antennal transcriptomics studies [94,95] but was later renamed *LdecOBP33* in a genome-wide analysis [33]. In a population genetics study using a linkage disequilibrium-based method (HapFLK), *LdecOBP33* was identified as being under selection, suggesting adaptive divergence between insecticide-susceptible and -resistant beetle populations. These findings indicate that *LdecOBP33* may be evolving specifically in response to insecticide pressures [96]. A recently published tissue-specific gene expression atlas for *Leptinotarsa decemlineata* reported high expression of *LdecOBP33* in the male fat body (https://cpb-atlas.uni-mainz.de/gene/display/LdNA_28773, accessed on 2 November 2025); however, antennae were not included in this dataset [97]. In contrast, our study demonstrates that *LdecOBP33* is highly expressed in the antennae, particularly in males (Figure 1B). The observed sex-biased expression pattern suggests that *LdecOBP33* may also play a role in mate recognition or other male-specific interactions. Further investigation is required to test this hypothesis.

## Figures and Tables

**Table 1:** Data representing binding of competitor ligands to LdecOBP33.

Category	Ligand Name	^a^ IC50 (µM)	Ki (µM)	^b^ LogP
Potato plant volatiles	Nonanal	7.29 ± 0.53	5.47	3.30
	(Z)-3-Hexenyl-butyrate	23.99 ± 2.05	18.01	2.70
	L-Linalool	48.26 ± 1.77	36.23	2.70
	Methyl salicylate	396.30 ± 19.97	297.54	2.50
	(E)-2-Hexenal	401.40 ± 16.29	301.37	1.50
	2-Phenylethanol	838.50 ± 39.54	629.54	1.36
	(E)-3-Hexen-1-ol	-	-	1.30
Plant products	D-Glucose	-	-	−2.60
Neonicotinoids	Clothianidin	94.51 ± 4.24	70.96	0.70
	Imidacloprid	262.80 ± 15.17	197.31	0.57
Organophosphates	Chlorpyrifos	45.81 ± 3.53	34.39	4.96
	Chlorpyrifos-methyl	63.67 ± 3.32	47.80	4.30
Carbamates	Carbaryl	-	-	2.36
Pyrethroids	Tetramethrin	29.07 ± 1.78	21.83	4.70

^a^: shows mean ± SE (*n* = 3); ^b^: indicates values obtained from PubChem database (accessed on 31 October 2025); -: indicates competitor ligands that did not display intrinsic binding affinity to LdOBP33.

## Data Availability

The original contributions presented in this study are included in the article/Supplementary Material. Further inquiries can be directed to the corresponding authors.

## Supplementary Materials

The following supporting information can be downloaded at https://www.mdpi.com/article/10.3390/insects16121259/s1, Figure S1: Phylogenetic relationships of Colorado potato beetle (CPB) odorant-binding proteins (OBPs) across different coleopteran species. Yellow (classic OBPs), green (ABPII classic OBPs), red (minus-C OBPs), and purple (ABPII minus-C OBPs) coloration indicates the class of OBPs. Color of label text indicates species (blue, *Diabrotica virgifera virgifera*; purple, *Tribolium castaneum*; light blue, *Collaphellus bowringi*; red, *Leptinotarsa decemlineata*; green, *Monochromatus alternatus*). Star indicates location of LdecOBP33. The phylogenetic tree was inferred using the maximum likelihood estimation method, with the LG + G + I model, using the MEGA11 software v11.0.13. The tree was visualized using the Figtree v1.4.4 software. Table S1: Protein sequences of insect OBP genes were used to construct a maximum likelihood (ML) phylogenetic tree. Sequences not listed with a complementary accession number are adapted from sequences from the prior literature that did not feature an accession number. Table S2: Primers used in this study.

## Abbreviations

The following abbreviations are used in this manuscript:

OBPs	Odorant-binding proteins
CPB	Colorado potato beetle
RNAi	RNA interference
dsRNA	Double-stranded RNA
PMSF	Phenylmethylsulfonyl fluoride
IPTG	Isopropyl β-d-1-thiogalactopyranoside
EDTA	Ethylenediaminetetraacetic acid
SDS-PAGE	Sodium dodecyl sulfate–polyacrylamide gel electrophoresis
