# Supplementary material for: Pleiotropic Function of Antenna-Specific Odorant-Binding Protein Links Xenobiotic Adaptation and Olfaction in Leptinotarsa decemlineata
Source: Insects. 2025 Dec 11;16(12):1259. doi: 10.3390/insects16121259 (PMC12733967; doi:10.3390/insects16121259)
Supplement: Supplementary file 1 [file insects-16-01259-s001.zip › insects-3993689-supplementary.pdf]

Support information

Pleiotropic function of antenna-specific odorant-binding protein links xenobiotic adaptation and olfaction in *Leptinotarsa decemlineata*

James A. Abendroth [a], Timothy W. Moural [a,1], Casey Cruse [a], Jonathan A. Hernandez [a], Michael Wolfin [a], Tom C. Baker [a], Andrei Alyokhin [b], Fang Zhu [a,c,1]

[a] *Department of Entomology, Pennsylvania State University, University Park, PA 16802, USA*

[b] School of Biology and Ecology, University of Maine, Orono, ME 04469, USA

[c] *Huck Institutes of the Life Sciences, Pennsylvania State University, University Park, PA 16802, USA*

[1] To whom correspondence should be addressed. Email address: fuz59@psu.edu; twm78@psu.edu

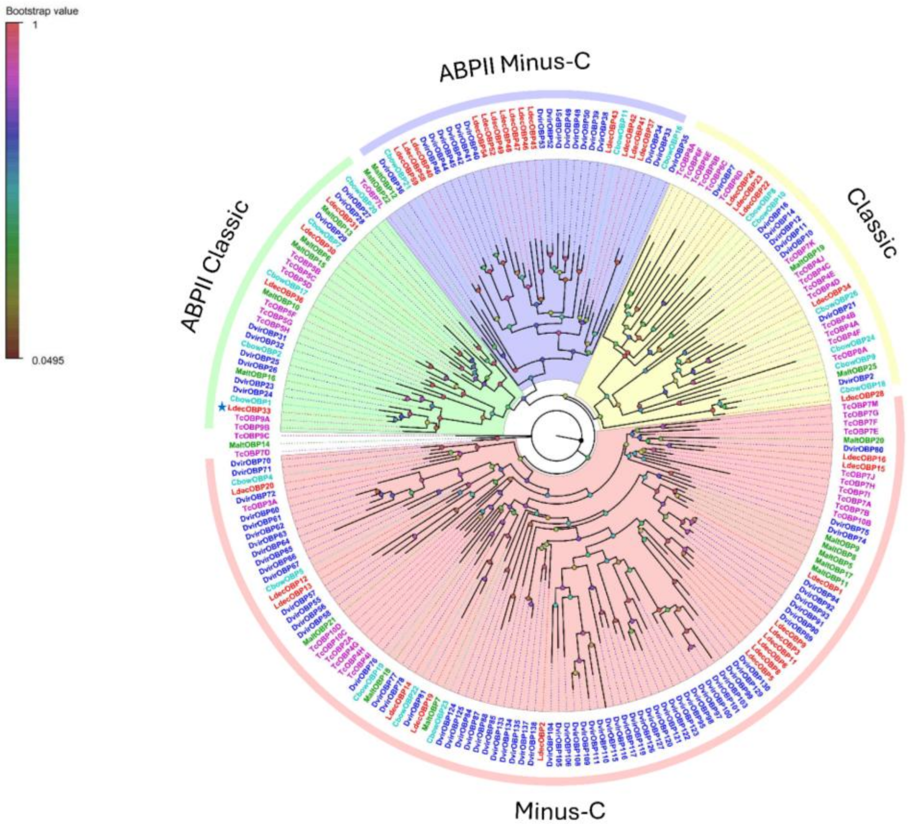

Figure S1. Phylogenetic relationships of Colorado potato beetle (CPB) odorant binding proteins (OBPs) across different coleopteran species. Yellow (classic OBPs), green (ABPII classic OBPs), red (minus-C OBPs), or purple (ABPII minus-C OBPs) coloration indicates the classes of OBPs. Color of label text indicates species (blue, *Diabrotica virgifera virgifera*; purple, *Tribolium castaneum*; light blue, *Collaphellus bowringi*; red, *Leptinotarsa decemlineata*; green, *Monochromatus alternatus*). Star denotes the location of LdecOBP33. The phylogenetic tree was inferred using the maximum likelihood estimation method, with the LG + G + I model, using MEGA11 software v11.0.13. The tree was visualized using Figtree v1.4.4 software.

Table S1. Protein sequences of insect OBP genes are used to construct a maximum likelihood (ML) phylogenetic tree. Sequences not listed with a complementary accession number are adapted from sequences from prior literature that did not feature an accession number.

Reference DOI	Name	Accession number	Species
10.1016/j.cbd.2014.05.001	MaltOBP5	AIX97020.1	*Monochomatus alternatus*
10.1016/j.cbd.2014.05.001	MaltOBP6	AIX97021.1	*Monochomatus alternatus*
10.1016/j.cbd.2014.05.001	MaltOBP7	AIX97022.1	*Monochomatus alternatus*
10.1016/j.cbd.2014.05.001	MaltOBP8	AIX97023.1	*Monochomatus alternatus*
10.1016/j.cbd.2014.05.001	MaltOBP9	AIX97024.1	*Monochomatus alternatus*
10.1016/j.cbd.2014.05.001	MaltOBP10	AIX97025.1	*Monochomatus alternatus*
10.1016/j.cbd.2014.05.001	MaltOBP11	AIX97026.1	*Monochomatus alternatus*
10.1016/j.cbd.2014.05.001	MaltOBP12	AIX97027.1	*Monochomatus alternatus*
10.1016/j.cbd.2014.05.001	MaltOBP13	AIX97028.1	*Monochomatus alternatus*
10.1016/j.cbd.2014.05.001	MaltOBP14	AIX97029.1	*Monochomatus alternatus*
10.1016/j.cbd.2014.05.001	MaltOBP15	AIX97030.1	*Monochomatus alternatus*
10.1016/j.cbd.2014.05.001	MaltOBP16	AIX97031.1	*Monochomatus alternatus*
10.1016/j.cbd.2014.05.001	MaltOBP17	AIX97032.1	*Monochomatus alternatus*
10.1016/j.cbd.2014.05.001	MaltOBP18	AIX97033.1	*Monochomatus alternatus*
10.1016/j.cbd.2014.05.001	MaltOBP19	AIX97034.1	*Monochomatus alternatus*
10.1016/j.cbd.2014.05.001	MaltOBP20	AIX97035.1	*Monochomatus alternatus*
10.1016/j.cbd.2014.05.001	MaltOBP21	AIX97036.1	*Monochomatus alternatus*
10.1016/j.cbd.2014.05.001	MaltOBP22	AIX97037.1	*Monochomatus alternatus*
10.1016/j.cbd.2014.05.001	MaltOBP25	AIX97019.1	*Monochomatus alternatus*
10.1186/s12864-015-2236-3	CbowOBP1	ALR72489.1	*Collaphellus bowringi*
10.1186/s12864-015-2236-3	CbowOBP2	ALR72490.1	*Collaphellus bowringi*
10.1186/s12864-015-2236-3	CbowOBP4	ALR72492.1	*Collaphellus bowringi*
10.1186/s12864-015-2236-3	CbowOBP5	ALR72493.1	*Collaphellus bowringi*
10.1186/s12864-015-2236-3	CbowOBP7	ALR72495.1	*Collaphellus bowringi*
10.1186/s12864-015-2236-3	CbowOBP8	ALR72496.1	*Collaphellus bowringi*
10.1186/s12864-015-2236-3	CbowOBP9	ALR72497.1	*Collaphellus bowringi*
10.1186/s12864-015-2236-3	CbowOBP10	ALR72498.1	*Collaphellus bowringi*
10.1186/s12864-015-2236-3	CbowOBP11	ALR72499.1	*Collaphellus bowringi*
10.1186/s12864-015-2236-3	CbowOBP16	ALR72504.1	*Collaphellus bowringi*
10.1186/s12864-015-2236-3	CbowOBP17	ALR72505.1	*Collaphellus bowringi*
10.1186/s12864-015-2236-3	CbowOBP18	ALR72506.1	*Collaphellus bowringi*
10.1186/s12864-015-2236-3	CbowOBP19	ALR72507.1	*Collaphellus bowringi*
10.1186/s12864-015-2236-3	CbowOBP20	ALR72508.1	*Collaphellus bowringi*
10.1186/s12864-015-2236-3	CbowOBP21	ALR72509.1	*Collaphellus bowringi*
10.1186/s12864-015-2236-3	CbowOBP22	ALR72510.1	*Collaphellus bowringi*
10.1186/s12864-015-2236-3	CbowOBP23	ALR72511.1	*Collaphellus bowringi*
10.1186/s12864-015-2236-3	CbowOBP24	ALR72512.1	*Collaphellus bowringi*
10.1186/s12864-015-2236-3	CbowOBP26	ALR72514.1	*Collaphellus bowringi*
10.1038/s41598-018-20154-1	LdecOBP1	*	*Leptinotarsa decemlineata*
10.1038/s41598-018-20154-1	LdecOBP2	*	*Leptinotarsa decemlineata*
10.1038/s41598-018-20154-1	LdecOBP3	*	*Leptinotarsa decemlineata*
10.1038/s41598-018-20154-1	LdecOBP5	*	*Leptinotarsa decemlineata*
10.1038/s41598-018-20154-1	LdecOBP6	*	*Leptinotarsa decemlineata*
10.1038/s41598-018-20154-1	LdecOBP8	*	*Leptinotarsa decemlineata*
10.1038/s41598-018-20154-1	LdecOBP9	*	*Leptinotarsa decemlineata*
10.1038/s41598-018-20154-1	LdecOBP11	*	*Leptinotarsa decemlineata*
10.1038/s41598-018-20154-1	LdecOBP12	*	*Leptinotarsa decemlineata*
10.1038/s41598-018-20154-1	LdecOBP13	*	*Leptinotarsa decemlineata*

10.1038/s41598-018-20154-1	LdecOBP14	*	*Leptinotarsa decemlineata*
10.1038/s41598-018-20154-1	LdecOBP15	*	*Leptinotarsa decemlineata*
10.1038/s41598-018-20154-1	LdecOBP16	*	*Leptinotarsa decemlineata*
10.1038/s41598-018-20154-1	LdecOBP19	*	*Leptinotarsa decemlineata*
10.1038/s41598-018-20154-1	LdecOBP20	*	*Leptinotarsa decemlineata*
10.1038/s41598-018-20154-1	LdecOBP22	*	*Leptinotarsa decemlineata*
10.1038/s41598-018-20154-1	LdecOBP23	*	*Leptinotarsa decemlineata*
10.1038/s41598-018-20154-1	LdecOBP24	*	*Leptinotarsa decemlineata*
10.1038/s41598-018-20154-1	LdecOBP28	*	*Leptinotarsa decemlineata*
10.1038/s41598-018-20154-1	LdecOBP30	*	*Leptinotarsa decemlineata*
10.1038/s41598-018-20154-1	LdecOBP31	*	*Leptinotarsa decemlineata*
10.1038/s41598-018-20154-1	LdecOBP33	XP_023021253	*Leptinotarsa decemlineata*
10.1038/s41598-018-20154-1	LdecOBP34	*	*Leptinotarsa decemlineata*
10.1038/s41598-018-20154-1	LdecOBP36	XP_023024287	*Leptinotarsa decemlineata*
10.1038/s41598-018-20154-1	LdecOBP37	*	*Leptinotarsa decemlineata*
10.1038/s41598-018-20154-1	LdecOBP40	*	*Leptinotarsa decemlineata*
10.1038/s41598-018-20154-1	LdecOBP41	*	*Leptinotarsa decemlineata*
10.1038/s41598-018-20154-1	LdecOBP42	*	*Leptinotarsa decemlineata*
10.1038/s41598-018-20154-1	LdecOBP43	*	*Leptinotarsa decemlineata*
10.1038/s41598-018-20154-1	LdecOBP45	*	*Leptinotarsa decemlineata*
10.1038/s41598-018-20154-1	LdecOBP46	*	*Leptinotarsa decemlineata*
10.1038/s41598-018-20154-1	LdecOBP47	*	*Leptinotarsa decemlineata*
10.1038/s41598-018-20154-1	LdecOBP48	*	*Leptinotarsa decemlineata*
10.1038/s41598-018-20154-1	LdecOBP49	*	*Leptinotarsa decemlineata*
10.1038/s41598-018-20154-1	LdecOBP52	*	*Leptinotarsa decemlineata*
10.1038/s41598-018-20154-1	LdecOBP54	*	*Leptinotarsa decemlineata*
10.1038/s41598-018-20154-1	LdecOBP58	*	*Leptinotarsa decemlineata*
10.1038/s41598-018-20154-1	LdecOBP59	*	*Leptinotarsa decemlineata*
10.1186/1471-2164-15-1141	TcOBP0A	**	*Tribolium castaneum*
10.1186/1471-2164-15-1141	TcOBP4A	**	*Tribolium castaneum*
10.1186/1471-2164-15-1141	TcOBP4B	**	*Tribolium castaneum*
10.1186/1471-2164-15-1141	TcOBP4C	**	*Tribolium castaneum*
10.1186/1471-2164-15-1141	TcOBP4D	**	*Tribolium castaneum*
10.1186/1471-2164-15-1141	TcOBP4E	**	*Tribolium castaneum*
10.1186/1471-2164-15-1141	TcOBP4F	**	*Tribolium castaneum*
10.1186/1471-2164-15-1141	TcOBP4J	**	*Tribolium castaneum*
10.1186/1471-2164-15-1141	TcOBP5B	**	*Tribolium castaneum*
10.1186/1471-2164-15-1141	TcOBP5C	**	*Tribolium castaneum*
10.1186/1471-2164-15-1141	TcOBP5D	**	*Tribolium castaneum*
10.1186/1471-2164-15-1141	TcOBP5F	**	*Tribolium castaneum*
10.1186/1471-2164-15-1141	TcOBP5G	**	*Tribolium castaneum*
10.1186/1471-2164-15-1141	TcOBP5H	**	*Tribolium castaneum*
10.1186/1471-2164-15-1141	TcOBP6B	**	*Tribolium castaneum*
10.1186/1471-2164-15-1141	TcOBP6C	**	*Tribolium castaneum*
10.1186/1471-2164-15-1141	TcOBP6D	**	*Tribolium castaneum*
10.1186/1471-2164-15-1141	TcOBP6E	**	*Tribolium castaneum*
10.1186/1471-2164-15-1141	TcOBP6F	**	*Tribolium castaneum*
10.1186/1471-2164-15-1141	TcOBP7D	**	*Tribolium castaneum*
10.1186/1471-2164-15-1141	TcOBP8A	**	*Tribolium castaneum*
10.1186/1471-2164-15-1141	TcOBP9A	**	*Tribolium castaneum*
10.1186/1471-2164-15-1141	TcOBP9B	**	*Tribolium castaneum*
10.1186/1471-2164-15-1141	TcOBP2A	**	*Tribolium castaneum*
10.1186/1471-2164-15-1141	TcOBP3A	**	*Tribolium castaneum*

10.1186/1471-2164-15-1141	TcOBP4G	**	*Tribolium castaneum*
10.1186/1471-2164-15-1141	TcOBP4H	**	*Tribolium castaneum*
10.1186/1471-2164-15-1141	TcOBP4I	**	*Tribolium castaneum*
10.1186/1471-2164-15-1141	TcOBP7A	**	*Tribolium castaneum*
10.1186/1471-2164-15-1141	TcOBP7B	**	*Tribolium castaneum*
10.1186/1471-2164-15-1141	TcOBP7E	**	*Tribolium castaneum*
10.1186/1471-2164-15-1141	TcOBP7F	**	*Tribolium castaneum*
10.1186/1471-2164-15-1141	TcOBP7G	**	*Tribolium castaneum*
10.1186/1471-2164-15-1141	TcOBP7H	**	*Tribolium castaneum*
10.1186/1471-2164-15-1141	TcOBP7I	**	*Tribolium castaneum*
10.1186/1471-2164-15-1141	TcOBP7J	**	*Tribolium castaneum*
10.1186/1471-2164-15-1141	TcOBP7K	**	*Tribolium castaneum*
10.1186/1471-2164-15-1141	TcOBP7L	**	*Tribolium castaneum*
10.1186/1471-2164-15-1141	TcOBP7M	**	*Tribolium castaneum*
10.1186/1471-2164-15-1141	TcOBP9C	**	*Tribolium castaneum*
10.1186/1471-2164-15-1141	TcOBP10B	**	*Tribolium castaneum*
10.1186/1471-2164-15-1141	TcOBP10C	**	*Tribolium castaneum*
10.1186/1471-2164-15-1141	TcOBP10D	**	*Tribolium castaneum*
10.1186/s12864-022-08990-y	DvirOBP2	***	*Diabrotica virgifera virgifera*
10.1186/s12864-022-08990-y	DvirOBP7	***	*Diabrotica virgifera virgifera*
10.1186/s12864-022-08990-y	DvirOBP10	***	*Diabrotica virgifera virgifera*
10.1186/s12864-022-08990-y	DvirOBP11	***	*Diabrotica virgifera virgifera*
10.1186/s12864-022-08990-y	DvirOBP12	***	*Diabrotica virgifera virgifera*
10.1186/s12864-022-08990-y	DvirOBP14	***	*Diabrotica virgifera virgifera*
10.1186/s12864-022-08990-y	DvirOBP16	***	*Diabrotica virgifera virgifera*
10.1186/s12864-022-08990-y	DvirOBP21	***	*Diabrotica virgifera virgifera*
10.1186/s12864-022-08990-y	DvirOBP23	***	*Diabrotica virgifera virgifera*
10.1186/s12864-022-08990-y	DvirOBP24	***	*Diabrotica virgifera virgifera*
10.1186/s12864-022-08990-y	DvirOBP25	***	*Diabrotica virgifera virgifera*
10.1186/s12864-022-08990-y	DvirOBP26	***	*Diabrotica virgifera virgifera*
10.1186/s12864-022-08990-y	DvirOBP27	***	*Diabrotica virgifera virgifera*
10.1186/s12864-022-08990-y	DvirOBP28	***	*Diabrotica virgifera virgifera*
10.1186/s12864-022-08990-y	DvirOBP29	***	*Diabrotica virgifera virgifera*
10.1186/s12864-022-08990-y	DvirOBP31	***	*Diabrotica virgifera virgifera*
10.1186/s12864-022-08990-y	DvirOBP32	***	*Diabrotica virgifera virgifera*
10.1186/s12864-022-08990-y	DvirOBP33	***	*Diabrotica virgifera virgifera*
10.1186/s12864-022-08990-y	DvirOBP34	***	*Diabrotica virgifera virgifera*
10.1186/s12864-022-08990-y	DvirOBP35	***	*Diabrotica virgifera virgifera*
10.1186/s12864-022-08990-y	DvirOBP36	***	*Diabrotica virgifera virgifera*
10.1186/s12864-022-08990-y	DvirOBP38	***	*Diabrotica virgifera virgifera*
10.1186/s12864-022-08990-y	DvirOBP39	***	*Diabrotica virgifera virgifera*
10.1186/s12864-022-08990-y	DvirOBP40	***	*Diabrotica virgifera virgifera*
10.1186/s12864-022-08990-y	DvirOBP41	***	*Diabrotica virgifera virgifera*
10.1186/s12864-022-08990-y	DvirOBP42	***	*Diabrotica virgifera virgifera*
10.1186/s12864-022-08990-y	DvirOBP44	***	*Diabrotica virgifera virgifera*
10.1186/s12864-022-08990-y	DvirOBP45	***	*Diabrotica virgifera virgifera*
10.1186/s12864-022-08990-y	DvirOBP46	***	*Diabrotica virgifera virgifera*
10.1186/s12864-022-08990-y	DvirOBP48	***	*Diabrotica virgifera virgifera*
10.1186/s12864-022-08990-y	DvirOBP49	***	*Diabrotica virgifera virgifera*
10.1186/s12864-022-08990-y	DvirOBP50	***	*Diabrotica virgifera virgifera*
10.1186/s12864-022-08990-y	DvirOBP51	***	*Diabrotica virgifera virgifera*
10.1186/s12864-022-08990-y	DvirOBP52	***	*Diabrotica virgifera virgifera*
10.1186/s12864-022-08990-y	DvirOBP53	***	*Diabrotica virgifera virgifera*

10.1186/s12864-022-08990-y	DvirOBP55	***	*Diabrotica virgifera virgifera*
10.1186/s12864-022-08990-y	DvirOBP56	***	*Diabrotica virgifera virgifera*
10.1186/s12864-022-08990-y	DvirOBP57	***	*Diabrotica virgifera virgifera*
10.1186/s12864-022-08990-y	DvirOBP58	***	*Diabrotica virgifera virgifera*
10.1186/s12864-022-08990-y	DvirOBP60	***	*Diabrotica virgifera virgifera*
10.1186/s12864-022-08990-y	DvirOBP61	***	*Diabrotica virgifera virgifera*
10.1186/s12864-022-08990-y	DvirOBP62	***	*Diabrotica virgifera virgifera*
10.1186/s12864-022-08990-y	DvirOBP63	***	*Diabrotica virgifera virgifera*
10.1186/s12864-022-08990-y	DvirOBP64	***	*Diabrotica virgifera virgifera*
10.1186/s12864-022-08990-y	DvirOBP65	***	*Diabrotica virgifera virgifera*
10.1186/s12864-022-08990-y	DvirOBP66	***	*Diabrotica virgifera virgifera*
10.1186/s12864-022-08990-y	DvirOBP67	***	*Diabrotica virgifera virgifera*
10.1186/s12864-022-08990-y	DvirOBP70	***	*Diabrotica virgifera virgifera*
10.1186/s12864-022-08990-y	DvirOBP71	***	*Diabrotica virgifera virgifera*
10.1186/s12864-022-08990-y	DvirOBP72	***	*Diabrotica virgifera virgifera*
10.1186/s12864-022-08990-y	DvirOBP74	***	*Diabrotica virgifera virgifera*
10.1186/s12864-022-08990-y	DvirOBP75	***	*Diabrotica virgifera virgifera*
10.1186/s12864-022-08990-y	DvirOBP76	***	*Diabrotica virgifera virgifera*
10.1186/s12864-022-08990-y	DvirOBP77	***	*Diabrotica virgifera virgifera*
10.1186/s12864-022-08990-y	DvirOBP78	***	*Diabrotica virgifera virgifera*
10.1186/s12864-022-08990-y	DvirOBP80	***	*Diabrotica virgifera virgifera*
10.1186/s12864-022-08990-y	DvirOBP81	***	*Diabrotica virgifera virgifera*
10.1186/s12864-022-08990-y	DvirOBP84	***	*Diabrotica virgifera virgifera*
10.1186/s12864-022-08990-y	DvirOBP85	***	*Diabrotica virgifera virgifera*
10.1186/s12864-022-08990-y	DvirOBP87	***	*Diabrotica virgifera virgifera*
10.1186/s12864-022-08990-y	DvirOBP88	***	*Diabrotica virgifera virgifera*
10.1186/s12864-022-08990-y	DvirOBP89	***	*Diabrotica virgifera virgifera*
10.1186/s12864-022-08990-y	DvirOBP90	***	*Diabrotica virgifera virgifera*
10.1186/s12864-022-08990-y	DvirOBP91	***	*Diabrotica virgifera virgifera*
10.1186/s12864-022-08990-y	DvirOBP92	***	*Diabrotica virgifera virgifera*
10.1186/s12864-022-08990-y	DvirOBP93	***	*Diabrotica virgifera virgifera*
10.1186/s12864-022-08990-y	DvirOBP94	***	*Diabrotica virgifera virgifera*
10.1186/s12864-022-08990-y	DvirOBP95	***	*Diabrotica virgifera virgifera*
10.1186/s12864-022-08990-y	DvirOBP97	***	*Diabrotica virgifera virgifera*
10.1186/s12864-022-08990-y	DvirOBP98	***	*Diabrotica virgifera virgifera*
10.1186/s12864-022-08990-y	DvirOBP99	***	*Diabrotica virgifera virgifera*
10.1186/s12864-022-08990-y	DvirOBP100	***	*Diabrotica virgifera virgifera*
10.1186/s12864-022-08990-y	DvirOBP101	***	*Diabrotica virgifera virgifera*
10.1186/s12864-022-08990-y	DvirOBP103	***	*Diabrotica virgifera virgifera*
10.1186/s12864-022-08990-y	DvirOBP104	***	*Diabrotica virgifera virgifera*
10.1186/s12864-022-08990-y	DvirOBP105	***	*Diabrotica virgifera virgifera*
10.1186/s12864-022-08990-y	DvirOBP106	***	*Diabrotica virgifera virgifera*
10.1186/s12864-022-08990-y	DvirOBP108	***	*Diabrotica virgifera virgifera*
10.1186/s12864-022-08990-y	DvirOBP109	***	*Diabrotica virgifera virgifera*
10.1186/s12864-022-08990-y	DvirOBP110	***	*Diabrotica virgifera virgifera*
10.1186/s12864-022-08990-y	DvirOBP111	***	*Diabrotica virgifera virgifera*
10.1186/s12864-022-08990-y	DvirOBP115	***	*Diabrotica virgifera virgifera*
10.1186/s12864-022-08990-y	DvirOBP116	***	*Diabrotica virgifera virgifera*
10.1186/s12864-022-08990-y	DvirOBP117	***	*Diabrotica virgifera virgifera*
10.1186/s12864-022-08990-y	DvirOBP119	***	*Diabrotica virgifera virgifera*
10.1186/s12864-022-08990-y	DvirOBP120	***	*Diabrotica virgifera virgifera*
10.1186/s12864-022-08990-y	DvirOBP121	***	*Diabrotica virgifera virgifera*
10.1186/s12864-022-08990-y	DvirOBP122	***	*Diabrotica virgifera virgifera*

10.1186/s12864-022-08990-y	DvirOBP123	***	*Diabrotica virgifera virgifera*
10.1186/s12864-022-08990-y	DvirOBP124	***	*Diabrotica virgifera virgifera*
10.1186/s12864-022-08990-y	DvirOBP125	***	*Diabrotica virgifera virgifera*
10.1186/s12864-022-08990-y	DvirOBP126	***	*Diabrotica virgifera virgifera*
10.1186/s12864-022-08990-y	DvirOBP127	***	*Diabrotica virgifera virgifera*
10.1186/s12864-022-08990-y	DvirOBP129	***	*Diabrotica virgifera virgifera*
10.1186/s12864-022-08990-y	DvirOBP130	***	*Diabrotica virgifera virgifera*
10.1186/s12864-022-08990-y	DvirOBP133	***	*Diabrotica virgifera virgifera*
10.1186/s12864-022-08990-y	DvirOBP134	***	*Diabrotica virgifera virgifera*
10.1186/s12864-022-08990-y	DvirOBP135	***	*Diabrotica virgifera virgifera*
10.1186/s12864-022-08990-y	DvirOBP137	***	*Diabrotica virgifera virgifera*
10.1186/s12864-022-08990-y	DvirOBP138	***	*Diabrotica virgifera virgifera*

**Leptinotarsa decemlineata* (LdecOBP) sequences adapted from: [33] Schoville SD, Chen YH, Andersson MN, Benoit JB, Bhandari A, Bowsher JH, et al. A model species for agricultural pest genomics: the genome of the Colorado potato beetle, Leptinotarsa decemlineata (Coleoptera: Chrysomelidae). Sci Rep. 2018;8:18.
****Tribolium castaneum*** (TcasOBP) sequences adapted from: [64] Dippel S, Oberhofer G, Kahnt J, Gerischer L, Opitz L, Schachtner J, et al. Tissue-specific transcriptomics, chromosomal localization, and phylogeny of chemosensory and odorant binding proteins from the red flour beetle Tribolium castaneum reveal subgroup specificities for olfaction or more general functions. BMC Genomics. 2014;15:14.
*** *Diabrotica virgifera virgifera* (DvirOBP) sequences adapted from: [62] Coates BS, Walden KKO, Lata D, Vellichirammal NN, Mitchell RF, Andersson MN, McKay R, Lorenzen MD, Grubbs N, Wang YH, Han J, Xuan JL, Willadsen P, Wang H, French BW, Bansal R, Sedky S, Souza D, Bunn D, Meinke LJ, Miller NJ, Siegfried BD, Sappington TW, Robertson HM. A draft Diabrotica virgifera virgifera genome: insights into control and host plant adaption by a major maize pest insect. BMC Genomics. 2023;24:19.

Table S2. Primers used in this study.

Name	Sequence (5' – 3')	Purpose
qLdOBP-AF-1	GCATCAGCATTGAAAACTGCAAAG	qRT-PCR
qLdOBP-AR-1	TGGTAAGAAGTAGTTCTCGGGATTATC	
dLdOBP-AF	TAATACGACTCACTATAGGGACTGGTAACAAACTATGCTG	RNA interference
dLdOBP-AR	TAATACGACTCACTATAGGGCTGCTATTCTTTCTGGAGC	
dGFP-F	TAATACGACTCACTATAGGGCCATCTCCTTCAAGGACG	
dGFP-R	TAATACGACTCACTATAGGGCTCAGGTAGTGGTTGTCG	
LdOBPdel-F	GAGATGACCGAAAAGCAG	Signal peptide deletion
LdOBPdel-R	GCTGCTGTGATGATGATG	
FullLdOBP1F	ATGAGATTTCCCATTTTCAT	Full length sequencing
FullLdOBP1R	TCATGGTAAGAAGTAGTTCT	
